# Study on the Probiotic Properties of Xinjiang-Characteristic Selenium-Enriched Lactic Acid Bacteria and the Distribution of Selenium Element

**DOI:** 10.3390/foods14203577

**Published:** 2025-10-21

**Authors:** Jingshu Chen, Yiming Jia, Huizi Chensheng, Lu Feng, Yawen Li, Tiantian Jian, Xue Han, Xiyue Niu, Qian Xu

**Affiliations:** 1 College of Food Science and Engineering, Tarim University, Alar 843300, China; chenjingshu0201@163.com (J.C.); jiayiming0420@163.com (Y.J.); cshz21066@163.com (H.C.); 17866502894@163.com (L.F.); 120060035@taru.edu.cn (X.N.); 2 Production & Construction Group Key Laboratory of Special Agricultural Products Further Processing in Southern Xinjiang, Tarim University, Alar 843300, China; 3 Analysis and Testing Center, Tarim University, Alar 843300, China; liyawen5226@163.com (Y.L.); jtt18168224512025@163.com (T.J.); 4 A Special Food and Drug and Biochemical Innovation Research Center, School of Chemistry and Chemical Engineering, Harbin Institute of Technology, Harbin 150001, China; xhan@hit.edu.cn

**Keywords:** selenium-enriched LAB, selenoproteins, selenium nanoparticles, probiotic properties, integrative omics

## Abstract

Selenium, a crucial trace element, has garnered significant attention in functional food development due to its effective conversion into organic forms. This study systematically investigated the selenium enrichment potential and metabolic regulation mechanisms of 50 lactic acid bacteria (LAB) strains from Xinjiang. Through sodium selenite tolerance tests, eight core strains with over 80% selenium enrichment were selected, with optimal enrichment conditions being a 37 °C temperature, 2% sodium chloride concentration, and pH of 6.0 in MRS medium. Functional tests demonstrated that selenium-enriched strains exhibited a significantly enhanced antioxidant capacity (demonstrated by DPPH and ABTS free radical scavenging activities) and improved gastrointestinal fluid tolerance, with strain No.41 showing the most outstanding performance. Scanning electron microscopy combined with energy-dispersive X-ray spectroscopy (SEM-EDX) revealed nanoscale selenium (1.34 keV) on cell surfaces. Further characterization showed that 68.94% of selenium was incorporated into selenoproteins, 7.61% into nucleic acids, and 7.02% into polysaccharides. Integrated metabolomic and proteomic studies have shown that selenium reduces the content of L-cysteine primarily by replacing sulfur and competing for key sites in cysteine-S-conjugate-β-lyase, S-adenosyl-L-cysteine hydrolase, and homocysteine synthase, ultimately leading to the synthesis of selenocysteine and selenomethionine. A correlation analysis between differential metabolites and proteins revealed selenium’s significant impacts on the metabolic networks of LAB, antioxidant mechanisms, energy metabolism, and membrane stability. This research provides new insights for developing selenium-enriched probiotics for functional dairy products and health supplements.

## 1. Introduction

Selenium (Se) is a crucial trace element known for its antioxidant, anticancer, and detoxifying properties, particularly in counteracting certain heavy metal ions [1]. Despite its importance, approximately one billion individuals globally are at risk of an inadequate selenium intake [2]. An insufficient intake of selenium can result in specific deficiency-related diseases, such as Keshan disease, and may lead to a compromised immunity [3] and thyroid dysfunction [4]. Conversely, an excessive intake of selenium can lead to toxicity, gastrointestinal disturbances, and imbalances in metabolism and minerals [5]. The recommended daily intake for adults ranges from 55 to 400 μg. Selenium occurs in various forms in nature, categorized into organic and inorganic selenium based on their binding forms. Organic selenium is not only considered safe but also exhibits a high bioavailability and pharmacological activities, including anticancer, antibacterial, antiviral, and antioxidant properties [6]. Inorganic selenium is characterized by its low bioavailability, and compounds like sodium selenite and sodium selenate are associated with a high genotoxicity [7]. Consequently, the conversion of inorganic selenium into organic selenium and the development of efficient and safe organic selenium carriers have emerged as prominent research areas in the fields of nutrition and health.

Microbial transformation represents a significant method for facilitating the conversion of inorganic selenium to organic selenium [8]. Research indicates that LAB, which function as probiotics, are extensively utilized in the conversion and enrichment of selenium [9]. These probiotics, commonly used in the food industry, are capable of reducing selenite to organic selenium and enhancing the bioaccessibility of selenium through their metabolic activities. Although excessive selenium can adversely affect the growth of LAB, an optimal concentration of selenium can significantly promote their growth [10]. Jingpeng et al. have demonstrated that LAB exhibit strong selenium tolerance and a high efficiency in selenium enrichment [11]. During the biotransformation of selenium, sodium selenite or selenate can be transported into bacterial cells via sulfate permease [12]. Research has demonstrated the presence of specific transporters and enzymes in certain strains of LAB [13]. These transporters and enzymes facilitate the conversion of inorganic selenium, such as sodium selenite, into organic selenium compounds, including selenoamino acids, through a series of enzymatic reactions [12]. Furthermore, in the food industry, the use of selenium-enriched LAB in the production of fermented dairy products, beverages, and other foods not only enhances the selenium content but also imparts unique flavors and functional properties. Owing to their superior probiotic functions and antioxidant effects, selenium-enriched LAB are also utilized in the development of health supplements internationally. Some health products containing selenium-enriched LAB are employed to improve human intestinal health, boost immunity, and provide other health benefits [14].

Although previous investigations have established the capacity of LAB to biotransform inorganic selenium compounds, systematic characterizations of the selenium enrichment potential, optimized cultivation parameters, and functional specificities of Xinjiang-endemic Lactobacillus strains remain inadequately addressed. Moreover, the fundamental molecular mechanisms underlying selenium–sulfur substitution in lactic acid bacteria (LAB) have not been fully elucidated through integrated multi-omics approaches. Additionally, whether the high-selenium soil environment in Xinjiang has led local LAB to evolve unique selenium metabolism pathways remains an unresolved scientific question requiring further exploration. Therefore, this study examines 50 strains of LAB isolated from the Xinjiang region. Initially, a preliminary screening is conducted to assess the strains’ tolerance to selenium and their probiotic properties. Subsequently, scanning electron microscopy, in conjunction with spectral detection technology, is employed to identify the distribution sites of selenium nanoparticles within the LAB cells. Following this, selenoproteins, selenopolysaccharides, and selenonucleic acids are extracted, and a systematic analysis is performed to investigate the distribution characteristics and content variations of selenium in these three types of biological macromolecules. Finally, metabolomic and proteomic techniques are utilized for an in-depth analysis of the metabolites of selenium-enriched LAB. This study aims to elucidate the biological distribution mechanism of selenium in lactic acid bacteria and its influence on metabolites, providing a theoretical basis and bacterial support for the development of selenium-rich functional food.

## 2. Materials and Methods

### 2.1. Experimental Reagents

The 50 strains of lactic acid bacteria were all isolated from sour milk in the homes of herdsmen in Altay, Tacheng, and Ili Kazak Autonomous Prefecture in Xinjiang. The experimental reagents used include the following: DNA extraction kit (TIANGEN, Tiangen Biochemical Technology (Beijing) Co., Ltd., Beijing, China); sodium selenite (Shanghai Aladdin Biochemical Technology Co., Ltd., Shanghai, China); DPPH and ABTS kits (Suzhou Grace Biotechnology Co., Ltd., Suzhou, China); MRS broth (Beijing Aoboxing Biotechnology Co., Ltd., Beijing, China); bovine bile salt (Shanghai Macklin Biochemical Technology Co., Ltd., Shanghai, China); 3,3′-diaminobenzidine (Shanghai Macklin Biochemical Technology Co., Ltd., Shanghai, China); disodium ethylenediaminetetraacetate (Tianjin Yongda Chemical Reagent Co., Ltd., Tianjin, China); pentanediol (Tianjin Yongsheng Fine Chemical Co., Ltd., Tianjin, China); and pepsin and trypsin (Beijing Solarbio Science & Technology Co., Ltd., Beijing, China).

### 2.2. Experimental Equipment

The TGL-20br high-speed refrigerated centrifuge, Shanghai Anting Scientific Instrument Factory (Shanghai, China); inductively coupled plasma optical emission spectrometer (ICP-OES: 5110), Agilent Technologies Co., Ltd. (Santa Clara, CA, USA); variable vacuum ultra-high resolution field emission scanning electron microscope, Thermo Fisher Scientific (Waltham, MA, USA); and ultra-high pressure liquid chromatography (H-Class) were purchased from Waters Technologies Co., Ltd. (Milford, MA, USA); the liquid chromatograph (Thermo EASY nLC 1000) and mass spectrometer (Thermo Scientific QE) were from Thermo Fisher Scientific (Waltham, MA, USA).

### 2.3. Experimental Method

#### 2.3.1. Exploring the Optimal Selenium Enrichment Concentration for LAB

LAB, stored at −80 °C, were reactivated and cultured at 37 °C for 24 h to reach the stationary phase. They were then propagated to a second generation, and their OD_600_ was measured. While the addition of sodium selenite in the early stage of lactic acid bacteria culture inhibits the growth of LAB, the addition at the logarithmic growth period promotes it [11]. Therefore, sodium selenite was added at 0, 20, 40, 60, 80, and 100 mg/mL during the logarithmic growth period of lactic acid bacteria. After another 24 h, color changes were observed, and OD_600_ was measured every 4 h.

#### 2.3.2. Exploring the Effects of Environmental Factors on the Growth of Selenium Content LAB

Selenium-enriched lactic acid bacteria were cultured at different temperatures (15 °C, 25 °C, 30 °C, 37 °C, 40 °C, 45 °C) under identical salt concentration and pH conditions for 24 h. Under constant temperature and pH conditions, the bacteria were cultured at varying salt concentrations (0%, 2%, 4%, 6%, 8%) for 24 h. When temperature and salt concentration remained constant, the bacteria were incubated at different pH levels (4.0, 5.0, 6.0, 7.0, 8.0) for 24 h. All experimental groups had their photometric absorbance at 600 nm (OD_600_) recorded every 4 h to monitor bacterial growth.

#### 2.3.3. Determination of Selenium Content in LAB by 3,3′-Diaminobenzidine Method

In accordance with the methodology outlined by Luo et al. [15], volumes of 0, 2, 4, 6, 8, and 10 mL of selenium standard solution were each made up to 10 mL. Subsequently, 4 mL of 5% EDTA-2Na solution and 2 mL of 0.5% 3,3′-diaminobenzidine solution were added to each. The mixtures were thoroughly combined and allowed to react for 30 min in the absence of light. Then, the pH was adjusted to neutral with ammonia, 4 mL of toluene was added to extract vigorously, the toluene layer was taken after standing, and the OD_425_ value was determined sequentially to draw the standard curve. The supernatant of the selenium-rich lactic acid bacteria cultured under optimal conditions was collected by centrifugation, and the process of standard curve was repeated. The selenium content in the supernatant, namely the residual inorganic selenium content, was calculated according to the standard curve. The selenium-rich rate was calculated according to Formula (1).

(1) $$\mathrm{Selenium-rich}\;\mathrm{rate}\;(\%)=(S_{1}-S_{2})/S_{1}\;\times \;100\%$$

In the formula, S_1_ is the total selenium content (µg) and S_2_ is the residual inorganic selenium content (µg).

#### 2.3.4. Determination of Probiotic Properties of Selenium-Enriched LAB

DPPH free radical scavenging capacity.

This was determined in accordance with the experimental protocol outlined in the DPPH free radical scavenging capacity assay kit.

ABTS free radical scavenging capacity.

This was determined in accordance with the experimental protocol outlined in the ABTS free radical scavenging capacity assay kit.

Analysis of the tolerance of selenium-enriched LAB in artificial gastric juice.

To prepare artificial gastric juice, as described by Hamdi et al. [16], adjust PBS buffer to pH 2.0, dissolve pepsin, and filter through a sterile membrane. Resuspend 1 mL of selenium-enriched LAB in PBS; add to 5 mL of the gastric juice, then add 1.5 mL of 0.5% NaCl, mix, and incubate at 37 °C. After 0 and 3 h, perform a 1:10 serial dilution with sterile saline, count viable bacteria, and calculate the 3 h survival rate using Formula (2).

(2) $$\mathrm{Survival}\;\mathrm{rate}\;(\%)=(\mathrm{lg}N_{3}/\mathrm{lg}N_{0})\;\times \;100\%$$

In the formula, N_3_ represents the number of viable LAB in artificial gastric juice after 3 h (CFU) and N_0_ represents the number of viable LAB in artificial gastric juice at 0 h (CFU).

Analysis of the tolerance of selenium-enriched LAB in artificial intestinal juice.

Preparation of Simulated Intestinal Fluid: Adjust the PBS solution to pH 8.0 with NaOH, then add trypsin and bovine bile salts. Mix 1 mL of selenium-enriched LAB with 5 mL of the fluid, homogenize, and incubate at 37 °C. Collect samples at 0 and 3 h to assess LAB viability and calculate survival rates using Formula (3).

(3) $$\mathrm{Survival}\;\mathrm{rate}\;(\%)=(\mathrm{lg}N_{3}/\mathrm{lg}N_{0})\;\times \;100\%$$

In the formula, N_3_ represents the number of viable LAB counted each time in artificial intestinal juice after 3 h (CFU) and N_0_ represents the number of viable LAB in artificial intestinal juice at 0 h (CFU).

#### 2.3.5. Exploring the Distribution of Selenium in LAB

Scanning electron microscopy combined with X-ray energy dispersive spectroscopy for characterizing selenium-enriched LAB.

The cultured selenium-enriched LAB were fixed in a 4 °C refrigerator with 2.5% glutaraldehyde, after which the samples were dehydrated using absolute ethanol. The dehydrated samples were freeze-dried at low temperature, and the morphology of the bacteria was observed via SEM. Subsequently, elemental analysis was performed using an X-ray energy dispersive spectrometer.

Selenium protein was extracted from selenium-rich lactic acid bacteria.

With slight modifications based on the method of Yuhan et al. [17], the LAB suspension was added to 50 mL of 0.25 M NaOH solution and subjected to ultrasonic treatment. The supernatant was collected, and the pH was adjusted to approximately 2.5 (±0.1) using 0.1 M HCl. After 4 h of sedimentation, the precipitate was obtained by centrifugation. A small amount of water was added to adjust the pH to neutral. The sample was pre frozen at minus 20 °C first, followed by preservation via freeze-drying.

Selenium polysaccharide was extracted from selenium-rich lactic acid bacteria.

With slight modifications based on the method described by Chen et al. [18], the LAB suspension was added to 50 mL of 0.25 M NaOH. After sonication and centrifugation, the supernatant was collected. The Sevag reagent was used to remove proteins from the supernatant, followed by a 12 h precipitation. The pH was adjusted to neutral with 0.1 M HCl, and alcohol precipitation was performed using 4 volumes of absolute ethanol. The precipitate was obtained through centrifugation, then pre-frozen at 20 °C below zero, and finally stored after freeze-drying.

Extract selenium nucleic acid from selenium-rich lactic acid bacteria.

This was conducted with reference to the experimental method in the DNA extraction kit.

Determination of selenium distribution by ICP-OES.

Submerge the microwave PTFE digestion tank in hot aqua regia, then carefully transfer the sample to an 80 mL PTFE digestion tank. Add a small amount of deionized water and nitric acid for digestion. Place the sample beaker and digestion tank into the microwave digestion instrument, and heat to 175 °C for 40 min. After digestion, cool the tank, transfer the solution to a 25 mL PTFE tube, dilute it to the marked volume, mix well, and prepare for testing. Use the same method for blank samples and measure their content.

#### 2.3.6. Metabolomics

LC analysis was performed on a Thermo Vanquish UHPLC system with an ACQUITY UPLC HSS T3 column (Waters, 1.8 μm, 2.1 × 100 mm) at 0.4 mL/min flow rate, 40 °C, and a 4 μL injection volume. Phase A: 0.1% formic acid; Phase B: 0.1% formic acid in acetonitrile. Mass spectrometry: Selections > 100 intensity for secondary data; range 50–1200, collision energy 30 eV, 15 spectra per cycle. ESI settings: GS1 60 Psi, auxiliary gas 60 Psi, curtain gas 35 Psi, 550 °C, spray voltage 5500 V (positive) or −4500 V (negative).

#### 2.3.7. Proteomics

After protease digestion, ZipTip C18 was used for desalting. Peptides were dissolved in 20 μL of 0.1% formic acid, vortexed, and centrifuged at 17,000× *g* and 4 °C for 20 min. The supernatant was transferred, and 3 μL was used for mass spectrometry. LC analysis was performed on a Thermo Vanquish UHPLC system with a PepMap RSLC C18 column (75 μm × 150 mm, 2 μm) at 0.4 mL/min flow rate, 40 °C, and a 4 μL injection volume. Phase A: 0.1% formic acid; Phase B: 0.1% formic acid in acetonitrile. Mass spectrometry settings: Primary resolution at 70,000, AGC target 3 × 10^6^, max injection time 60 ms, scan range 300–1400 *m*/*z*; secondary resolution at 17,500, AGC target 5 × 10^4^, max injection time 80 ms, TopN fragmentation (*N* = 20), NCE 27.

#### 2.3.8. Data Analysis

For proteomics, the PEAKS (Version 13) software was used for database searching. For metabolomics, based on the search of primary and secondary spectra, a self-integrated database incorporating Metlin, MassBank, MoNA, and HMDB (Version V6.0) was used to obtain identification results. For data analysis, SPSS (Version 2021), Origin (Version 2024), Graphpad Prism 9.5, and Metaboanalyst 6.0 were employed for data analysis and graphing. All experiments were performed with three biological repeats.

## 3. Results and Discussion

### 3.1. Tolerance of LAB to Selenium

**Figure 1:**
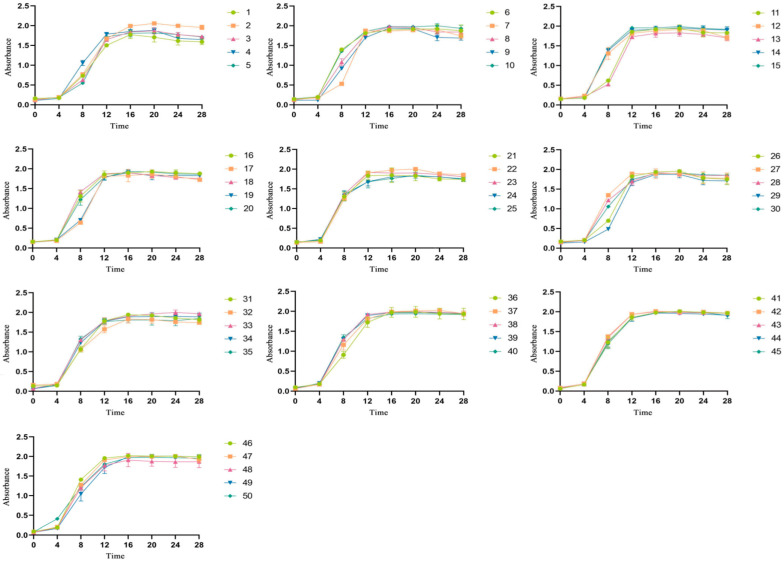
Growth curves of 50 LAB strains.

Upon measuring the optical density at 600 nm (OD_600_), it was determined that LAB strains 1 through 50 had entered the logarithmic growth phase by the 4 h mark (see Figure 1). At this stage, the MRS medium with varying concentrations of sodium selenite was added, and the cultures were incubated for an additional 24 h. Table 1 illustrates the resulting cell coloration, which progressively turned redder with increasing sodium selenite levels, indicating a positive correlation. Previous studies [11] have shown that LAB convert some selenium into selenocysteine, while the remainder is reduced to elemental selenium, resulting in the observed red color. The redness of the bacterial cells in this experiment indicates that an increase in the mass concentration of sodium selenite correlates with a gradual increase in the conversion of elemental selenium by the bacterial cells. When the selenium enrichment rate is fixed, the total amount of selenium enrichment remains constant. Consequently, as the transformation of elemental selenium increases, the synthesis of organic selenium by the bacterial cells decreases [19]. To optimize probiotic effects and enhance absorption, it is advisable to select a selenium concentration that minimally affects the color of bacterial cells. Red nano-selenium can impart a metallic taste, which may impact the fermentation of LAB [20]. Therefore, choosing the appropriate selenium concentration is essential. At the ideal concentration, the growth of LAB is not impeded, and selenium enrichment and conversion are optimized. In the study, a pink color signified the optimal concentration. With the exception of strains 3, 9, 13, 21, 28, 29, 32, and 35, which exhibited a pink color at 20 μg/mL, all other strains tolerated selenium optimally at 40 μg/mL.

### 3.2. Determination Results of Selenium Enrichment Rate

**Figure 2:**
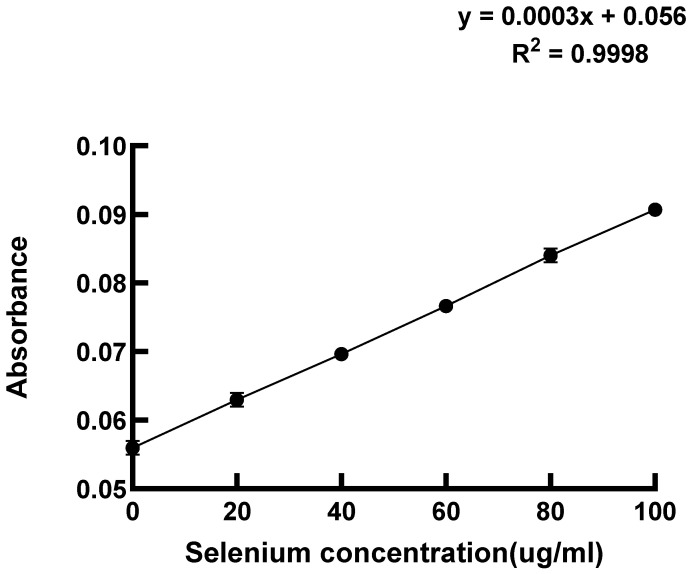
Standard curve for selenium enrichment rate.

Based on the optimal selenium enrichment concentrations identified for various strains in Table 1, the selenium enrichment rates were assessed post-cultivation, as depicted by the standard curve in Figure 2. The selenium standard curve shown in Figure 2 was fitted linearly (regression equation: y = 0.0003x + 0.056, R^2^ = 0.9998). The high R^2^ value (>0.995) of this linear model ensures the reliability of the quantitative results.

**Figure 3:**
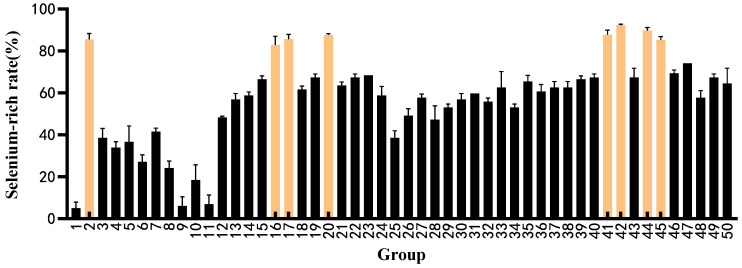
Selenium enrichment rate of strains under optimal selenium enrichment conditions (Yellow is the strain with high selenium content).

The selenium enrichment rates for these 50 strains were evaluated at their respective optimal selenium concentrations, with the findings illustrated in Figure 3. Among these strains, eight (specifically strains 2, 16, 17, 20, 41, 42, 44, and 45) exhibited selenium enrichment rates exceeding 80%, outperforming the others. Consequently, these eight strains have been selected for subsequent experiments focusing on probiotic properties.

### 3.3. Growth Status of 8 Selenium-Enriched LAB Strains Under Different Temperatures

**Figure 4:**
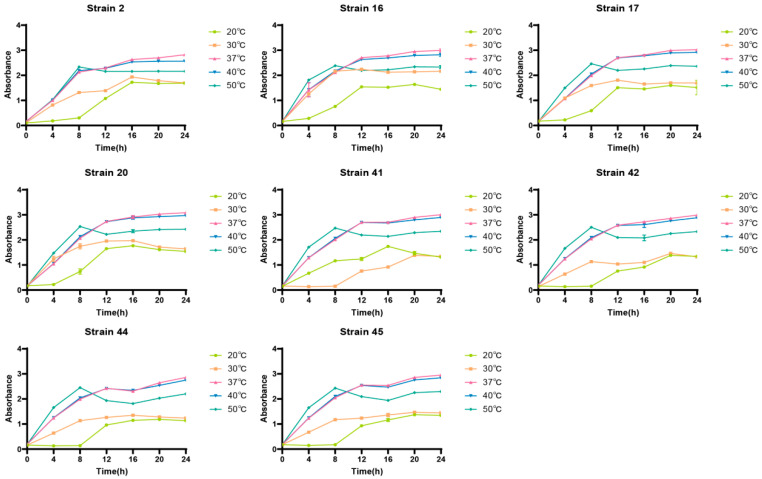
Growth status of selenium-enriched LAB at different temperatures.

From the growth status under different temperatures, it was found that, when the temperature reached 37 °C, the absorbance of the eight strains reached the highest during the stationary growth phase. Moreover, excessively low or high temperatures would inhibit the growth of LAB, as shown in Figure 4.

### 3.4. Growth Status of Selenium-Enriched LAB Under Different Salt Concentration Conditions

**Figure 5:**
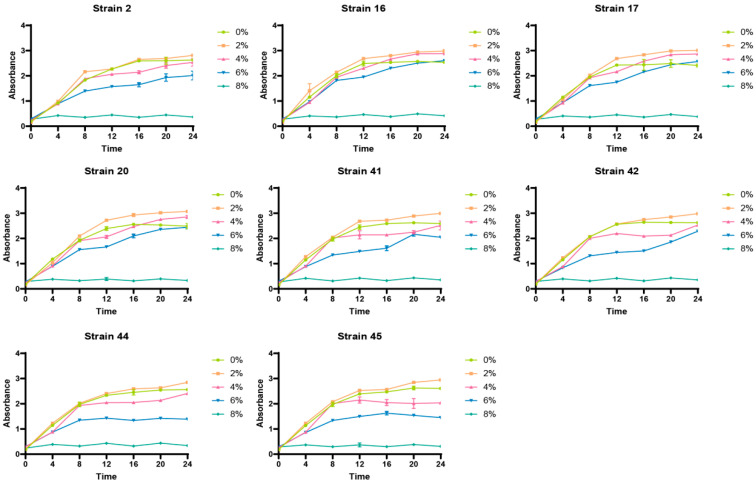
Growth status of selenium-enriched LAB under different salt concentrations.

The analysis of different salt concentrations showed that, when the salt concentration reached 2%, the absorbance of the eight strains peaked during the stationary growth phase, and excessively high salt concentrations would inhibit the growth of LAB, as shown in Figure 5.

### 3.5. Growth Status of Selenium-Enriched LAB Under Different pH Conditions

**Figure 6:**
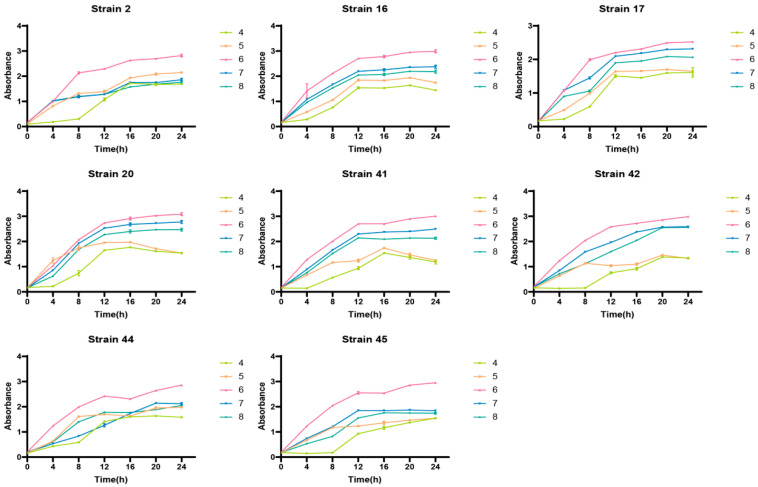
Growth status of selenium-enriched LAB under different pH conditions.

It was found through the analysis of different initial pH values that the optimal pH for the growth and development of selenium-enriched LAB is 6, and excessively high or low initial pH values will affect their growth, as shown in Figure 6.

In summary, the optimal growth conditions for selenium-enriched LAB are a temperature of 37 °C, a salt concentration of 2%, and an initial pH value of 6 in the culture medium.

### 3.6. Results of DPPH and ABTS Free Radical Scavenging Capacities

**Figure 7:**
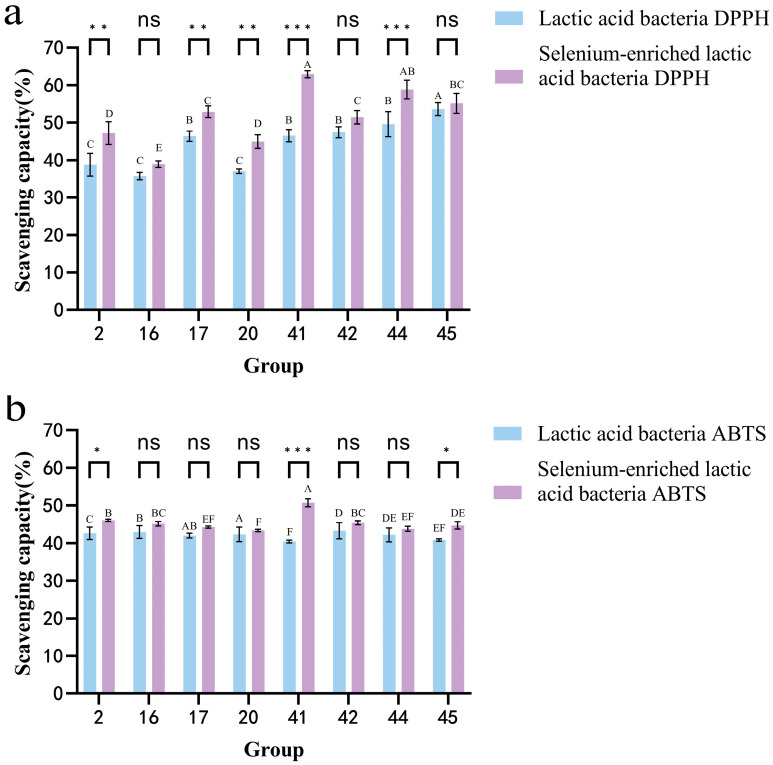
(**a**) DPPH and ABTS free radical scavenging capacity of LAB before and after selenium enrichment. (**b**) ABTS free radical scavenging capacity of LAB before and after selenium enrichment. Figure legend: Different uppercase letters indicate significant differences between different strains (*p* < 0.05); * indicates significant differences between the same strain before and after selenium enrichment (*p* < 0.05), ** (*p* < 0.01) and *** (*p* < 0.001) indicate greater significant differences; ns indicates no significance.

Figure 7 demonstrates that selenium-enriched strains have improved DPPH and ABTS free radical scavenging capacities compared to non-enriched strains. Notably, strain 41 showed significantly higher scavenging abilities than other selenium-enriched strains (*p* < 0.05), with increases of 14.06% and 10.27% for DPPH and ABTS, respectively, highlighting selenium’s role in boosting LAB’s antioxidant capacity [21]. Previous studies support this, showing that Bifidobacterium animalis’s antioxidant activity rises with selenium concentration [22]. Additionally, Liu et al. found that kiwi juice fermented with selenium-rich strains showed a higher antioxidant capacity [23].

### 3.7. Analysis of the Survival Rate of LAB in Artificial Gastric Juice and Intestinal Juice Before and After Selenium Enrichment

**Figure 8:**
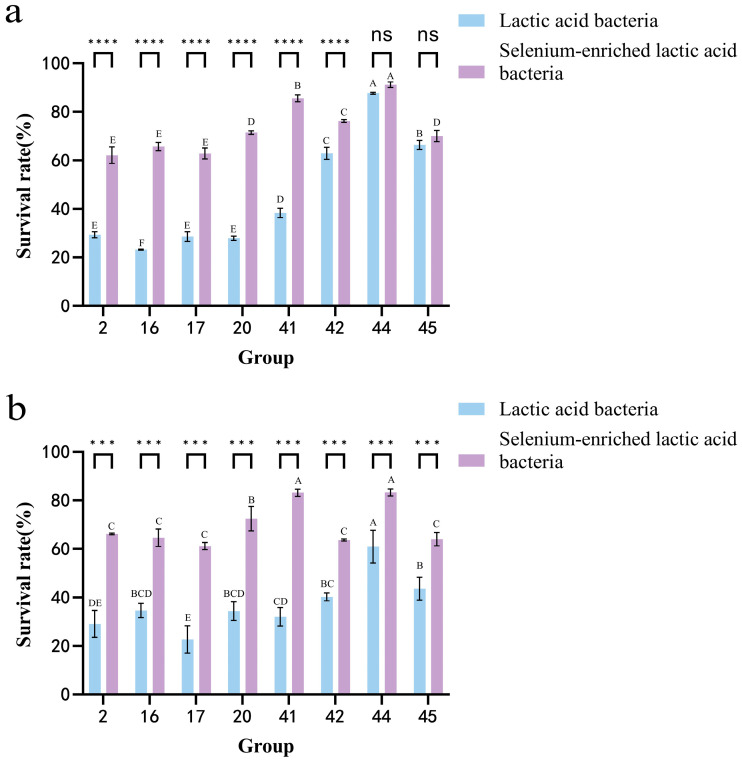
(**a**) Intestinal juice tolerance of LAB before and after selenium enrichment. (**b**) Gastric juice tolerance of LAB before and after selenium enrichment. Different uppercase letters indicate significant differences between different strains (*p* < 0.05); *** (*p* < 0.001) indicates significant differences between the same strain before and after selenium enrichment, **** (*p* < 0.0001) indicates greater significant differences; ns indicates no significance.

Figure 8 shows that selenium-enriched LAB have higher survival rates in gastric and intestinal juices after 3 h compared to non-enriched strains. In gastric juice, strain 44 had a high survival rate, but selenium enrichment significantly boosted the survival rates of strains 16, 20, and 41 by 42.56%, 43.59%, and 47.26%, respectively. Although selenium enrichment also improved strain 44’s survival, it was less pronounced. In intestinal juice, strain 44 again showed a high survival, but selenium-enriched strain 41’s survival increased significantly to 83.17%, a 51.13% improvement. Overall, selenium enrichment enhances the survival of LAB in both juices. This conclusion is consistent with the study by MARTINEZ et al. [24], who compared selenium-enriched and non-selenium-enriched Lactobacillus brevis CRL 2051 after fermenting fruit and vegetable juices. Under simulated gastrointestinal digestion, the selenium-enriched Lactobacillus brevis exhibited a higher survival rate.

### 3.8. Scanning Electron Microscope (SEM) Spectroscopy

**Figure 9:**
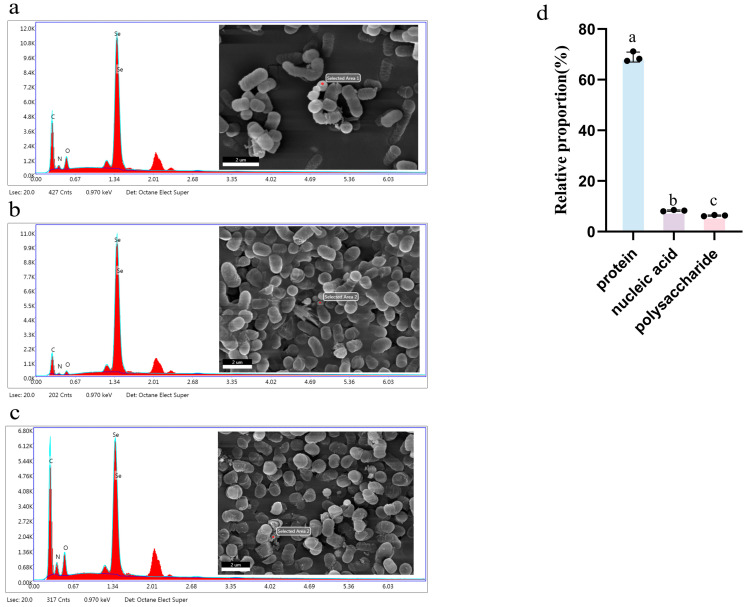
(**a**) Characterization diagram of LAB at a selenium enrichment concentration of 40 μg/mL. (**b**) Characterization diagram of LAB at a selenium enrichment concentration of 60 μg/mL. (**c**) Characterization diagram of LAB at a selenium enrichment concentration of 100 μg/mL. (**d**) Distribution of selenium element within cells. Different lowercase letters indicate significant differences between strains (*p* < 0.05).

In experiments, significant differences were observed in strain 41 before and after selenium enrichment (*p* < 0.05). Post-enrichment, field emission scanning electron microscopy and X-ray spectroscopy were used to analyze its cell structure and chemical composition. As shown in Figure 9a, low selenium concentrations resulted in tiny spherical particles on and around the cells. Elemental analysis using an EDX spectrometer detected selenium characteristic peaks at 1.34 keV, indicating selenium nanoparticles (Se NPs). Similarly, Averina et al. [22] found nano-selenium microspheres around selenium-enriched Lactobacillus brevis. This is because LAB can reduce inorganic selenium to elemental selenium (nano-selenium). The scanning analysis results confirmed the existence of elemental selenium, indicating that the selenium-enriched bacteria in the experiment did convert inorganic selenium. Interestingly, when the concentration reached 60 μg/mL, it was found that the selenium particles excreted by the cells became significantly larger (Figure 9b). This is because the Ostwald ripening mechanism is at work [25,26,27], whereby initial small selenium particles aggregate into large selenium nanoparticles. At a concentration of 100 μg/mL, some lactobacilli ruptured, and selenium was found in the debris (Figure 9c). This suggests that high selenium levels can inhibit lactobacilli growth, aligning with previous growth curve findings. Selenium can exist inside cells or convert into nanoparticles on cell surfaces. The excellent in vitro properties of selenium-enriched bacteria may be linked to these nano-selenium particles and converted organic selenium, as supported by antioxidant and tolerance tests.

### 3.9. Distribution of Selenium Element

As shown in Figure 9d, among the organic selenium components, protein accounts for 68.94 ± 1.98%, nucleic acid accounts for 7.61 ± 0.63%, and polysaccharide accounts for 7.02 ± 0.42%. It was observed that there were significant differences in the distribution of selenium among different components (*p* < 0.05), with most of the selenium present in proteins. This is consistent with the study on selenium enrichment in Lactobacillus paracasei, indicating that organic selenium tends to be incorporated into proteins in the form of selenoproteins [20]. Selenoproteins play important roles in processes such as anti-inflammatory responses [28], redox balance [29], ROS scavenging [30], promotion of thyroid metabolism [31], and antivirus and anticancer responses. Ahmed et al. found that Se NPs showed certain antibacterial activity against a variety of pathogens and MCF-7 and A549 tumor cell lines [1]. Blinova et al. also found that Se NPs have potential antibacterial and bactericidal properties against *Escherichia coli*, *Micrococcus luteus* and *Mucor* [32]. These findings highlight the ability of LAB to convert inorganic selenium into organic forms, emphasizing the potential of LAB in the selenium enrichment process.

### 3.10. Dntargeted Metabolomics

**Figure 10:**
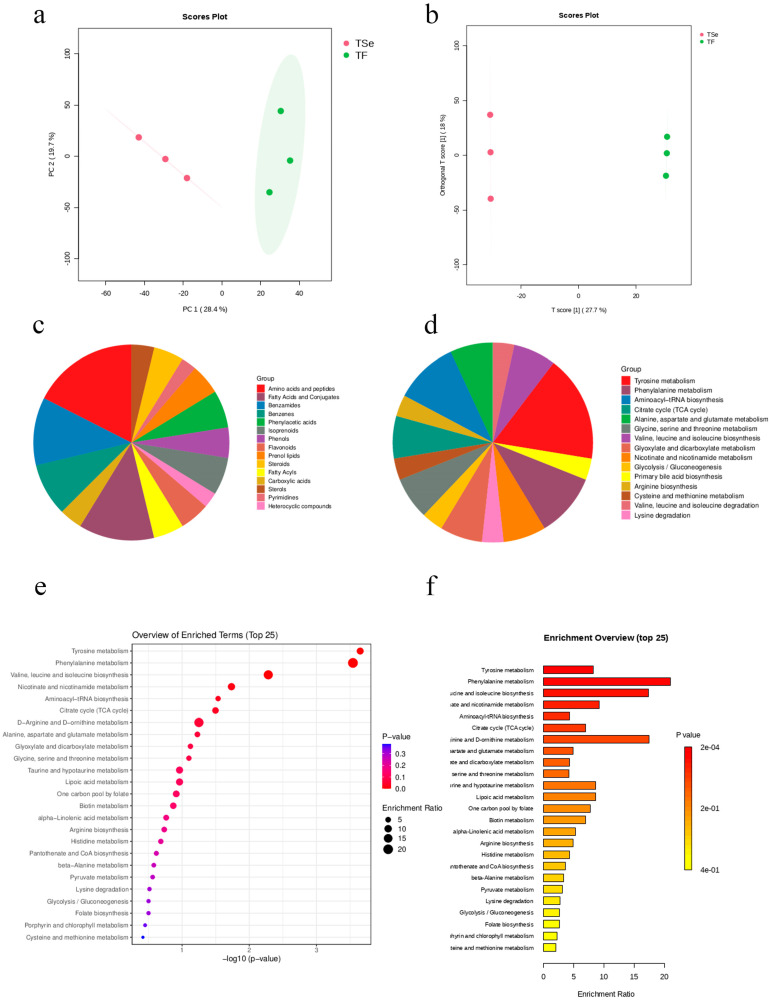
(**a**) PCA score plot. (**b**) Partial Least Squares (PLS) score plot. (**c**) Pie chart of differential metabolite classification. (**d**) Pie chart of enriched pathway classification for differential metabolites. (**e**) Bubble chart of differential metabolite enrichment results. (**f**) Bar chart of differential metabolite enrichment results.

To investigate metabolite changes in LAB before and after selenium enrichment, untargeted metabolomics analysis using LC-MS/MS was conducted. This analysis identified 3211 compounds in positive mode, with organic heterocyclic compounds being the most abundant (21.40%), followed by lipids (19.96%), benzene compounds (14.80%), and organic acids (14.54%). In negative mode, 1311 compounds were identified, with lipids (17.70%), organic acids (17.32%), benzene compounds (16.32%), and organic heterocyclic compounds (15.64%) being prominent. The PCA (Figure 10a) and OPLS-DA (Figure 10b) score plots showed an interpretability of 48.1% and 45.7%, respectively. The TF group (non-selenium-enriched LAB metabolites) and TSe group (selenium-enriched LAB metabolites) showed distinct differences. In metabolomics, identifying differential metabolites is crucial for understanding biological changes. Using the criteria of log2FC ≥ 1, *p*-value < 0.05, and VIP value ≥ 1, 491 differential metabolites were identified. The TSe group had 324 downregulated and 167 upregulated metabolites compared to the TF group. After matching with the HMDB database, 80 differential metabolites were found, with amino acids and peptides making up 18% and fatty acids and conjugates 13%. The addition of selenium mainly influences amino acid and fatty acid metabolism [33]. Metabolic pathway enrichment analysis, matched with the KEGG database, highlights the impacts on pathways such as tyrosine, phenylalanine, and aminoacyl-tRNA biosynthesis, among others [34]. This suggests that selenium affects amino acid and carbohydrate metabolism. Amino acids are known to benefit the immune and digestive systems [35]. They can support intestinal and overall body health, boost immune function, provide antioxidant benefits, speed up wound healing, and promote cardiovascular, liver, and blood sugar health [35,36]. Analysis showed significant metabolic pathway differences between the TF and TSe2 groups, including tyrosine and phenylalanine metabolism, valine, leucine, and isoleucine biosynthesis, nicotinate and nicotinamide metabolism, aminoacyl-tRNA biosynthesis, the TCA cycle, and arginine metabolism.

Studies have indicated that LAB can convert inorganic selenium into selenium-containing amino acids such as selenocysteine and selenomethionine [9], mainly due to the similar chemical properties of selenium and sulfur, where selenium can replace sulfide to combine with serine. Under normal conditions, cysteine synthetase synthesizes cysteine by catalyzing the reaction of sulfide with serine as the substrate through its synergistic interaction with serine acetyltransferase. However, in environments containing selenium, inorganic selenium competes for binding to the active center of cysteine synthetase, leading to the formation of selenocysteine. In the serine-to-cysteine metabolic pathway, three primary pathways have been identified: (1) Under the catalytic action of serine hydrolyase, L-serine reacts with L-homocysteine to form L-cystathionine. Subsequently, L-cystathionine is further converted into L-cysteine and 2-oxobutyric acid ester through the action of L-cysteine decarboxylase. (2) L-serine is converted into O-acetyl-L-serine with the participation of L-acetyl-CoA. This product then reacts with hydrogen sulfide under the action of cysteine synthetase to form L-cysteine and acetate. (3) L-methionine is transformed into S-adenosyl-L-methionine via methionine adenosyltransferase, subsequently generating S-adenosyl-L-homocysteine. S-adenosyl homocysteine is converted into S-ribosyl-L-homocysteine by S-ribosyl-L-homocysteine hydrolase. This compound is further processed into L-homocysteine through S-ribosyl homocysteine lyase. Finally, L-homocysteine undergoes serine hydrolysis to become L-cystathionine, which is ultimately cleaved into L-cysteine by L-cystathionine lysin. The metabolic analysis revealed that L-cysteine levels decreased significantly in TSe, while serine showed no significant changes. Since both serine and methionine conversion pathways to cysteine involve S-element participation, it remains unclear which specific pathway selenium substitutes for the S element to combine with serine or methionine to form selenocysteine. The mechanism of selenium enrichment is not yet established in the aspartate-to-methionine metabolic pathway. (4) L-aspartate is converted into L-4-phosphoadenine via L-aspartate-4-phosphatase. This product undergoes oxidoreductase action to generate L-aspartate-4-seminaldehyde, then aspartate-semialdehyde reductase to L-high serine. Subsequently catalyzed by succinyl-CoA, it forms coenzyme A-10-succinyl-L-high serine. This compound converts to L-cystathione with L-cysteine through lysylase action, then transforms into L-thyrosine via cysteine-S-conjugated β-ligase, ultimately producing L-methionine under transferase activity. The metabolite analysis showed that L-cysteine was significantly reduced while methionine content remained almost unchanged, indicating that selenium mainly affected the metabolic pathway of cysteine synthesizing methionine, which ultimately led to the production of selenomethionine. However, since sulfides were also involved in this pathway, it is not certain whether selenium affects this pathway.

Overall, selenium exerts significant effects on amino acids, fatty acids, and carbohydrate metabolism by regulating LAB metabolic pathways. These are consistent with the research of Peng et al. [37]. Particularly, through disrupting amino acid metabolism, it impacts bacterial growth and functionality. Selenium supplementation significantly reduces key metabolites like pyruvate, leucine, and aspartic acid, inhibits related synthase activity, and decreases certain metabolite accumulation. This is consistent with the conclusion of Wang et al. [34]. LAB utilize the chemical similarity between selenium and sulfur to convert inorganic selenium into selenocysteine and selenomethionine, mechanisms linked to cysteine synthesis and methionine metabolism: all three cysteine synthesis pathways involve sulfur [9,11]. Although selenium is hypothesized to competitively bind cysteine synthetase to substitute sulfur, its specific interaction with serine to form selenocysteine remains unclear. In the aspartic acid-to-methionine conversion pathway, cysteine levels decrease markedly while methionine content stays constant, suggesting selenium may influence cysteine-to-methionine conversion to produce selenomethionine. However, due to sulfide involvement in this pathway, its exact mechanism remains undefined. Therefore, proteomic analysis of enzymes in these metabolic pathways is required to clarify the selenium enrichment mechanisms within bacterial systems.

### 3.11. Proteomics

**Figure 11:**
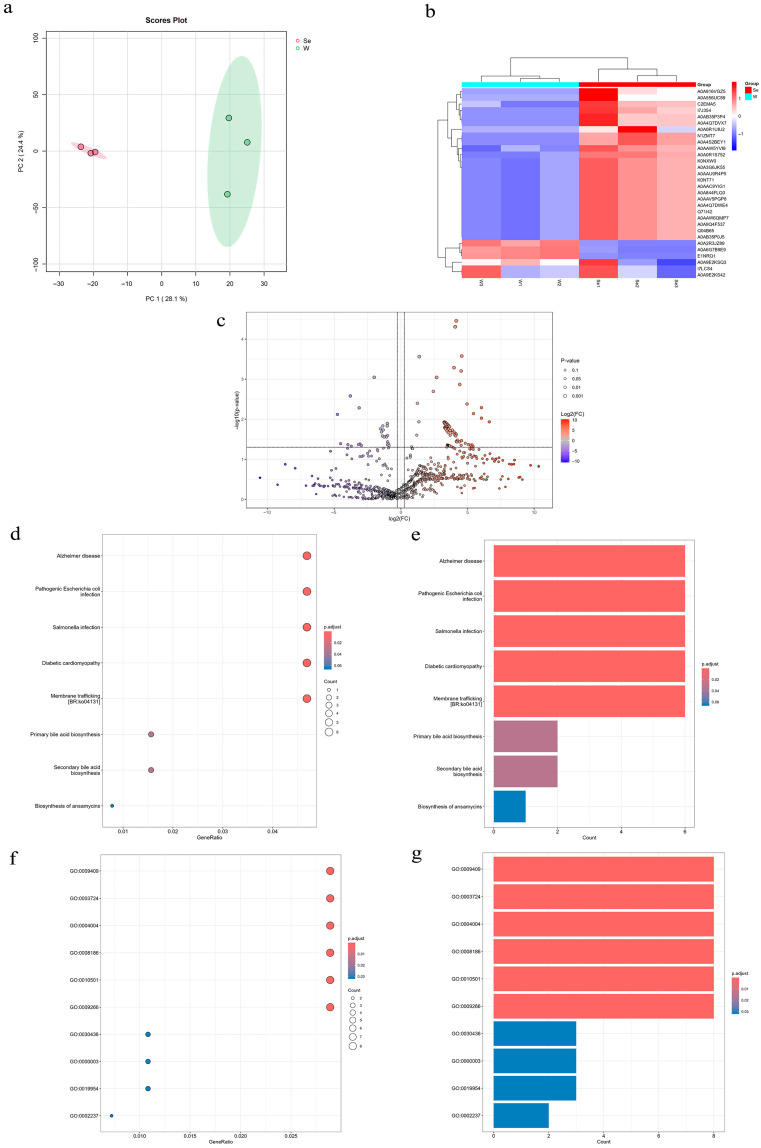
(**a**) PCA score plot. (**b**) Clustering heatmap of differential proteins. (**c**) Protein volcano plot. (**d**) Bubble plot of differential protein KEGG enrichment. (**e**) Bar plot of differential protein KEGG enrichment. (**f**) Bubble plot of differential protein GO enrichment. (**g**) Bar plot of differential protein GO enrichment.

In order to further elucidate the effects of selenium on enzymes and proteins in metabolomics on metabolites and metabolic pathways, LC-MS/MS proteomics was used to further identify the changes in metabolites in LAB and selenium-enriched LAB. After data standardization and normalization, a normal distribution was observed. The PCA score plot (Figure 11a) showed that PC1 and PC2 explained 52.5% of the variance. Differential proteins between TF and TSe were identified using fold change analysis and a T-test, resulting in 290 proteins after removing missing values, as depicted in the volcano plot (Figure 11c). A heatmap was generated through cluster analysis based on the expression levels of the top 30 differential proteins with the smallest *p*-values (Figure 11b), revealing that the main categories were aminoacyl-tRNA synthetases and transporters. Selenium primarily affects LAB by influencing nucleic acids and proteins. Selenocysteine can replace cysteine, forming selenium–sulfur bonds, and it can also trigger transporter expression in these bacteria. The KEGG enrichment analysis of differentially expressed proteins revealed that the main pathways affected include Alzheimer’s disease, pathogenic Escherichia coli infection, Salmonella infection, diabetic cardiomyopathy, and transmembrane transport, as shown in Figure 11d,e. These pathways differ significantly in LAB before and after selenium enrichment. Previous experiments have indicated that selenoproteins account for the majority of selenium-enriched LAB, and studies have shown that selenoproteins play an important role in antibacterial activity, antiviral activity [28], and transmembrane transport [38]. GO enrichment analysis was conducted on differential proteins using the Gene Ontology (GO) system, which classifies gene attributes into biological processes (BP), cellular components (CC), and molecular functions (MF). The analysis involved using the species’ proteins as a background list and the differential proteins as a candidate list. A hypergeometric distribution test calculated the *p*-value to determine significant enrichment in the candidate list, followed by FDR correction. The results are shown in Figure 11f,g. In selenium-enriched LAB, the biological processes primarily involving proteins include terpenoid biosynthesis, terpenoid backbone biosynthesis, RNA synthesis, ATPase activity, vesicle-mediated transport, and response to chemical stimuli. In the process of terpenoid biosynthesis, selenium may play a promoting role, and terpenoids can insert into cell membranes to regulate membrane fluidity and stability [39]. In the process of terpenoid backbone biosynthesis, selenium mainly functions as a catalyst, catalyzing the generation of terpenoid backbones such as chiral organic molecules derived from monoterpenes [40]. In the process of selenium binding to RNA, selenium can regulate the translation rate of RNA by affecting RNA-binding proteins. In the study by Alain Lescure et al., it was demonstrated that the binding of human SBP2 to SECIS RNA is stimulated by selenoprotein-specific elongation translation factors [41]. The effect of selenium on ATPase activity is mainly due to the fact that the synthesis of terpenoids requires a large amount of ATP consumption. Selenium maintains the balance between ATP and ADP by enhancing the activity of ATPase, and ensures the energy demand for terpenoid synthesis [42]. The response of selenium to chemical stimuli is mainly reflected in its inhibitory effect on viruses, inflammation, and pathogenic bacteria [43].

In metabolomic analysis, the impact of selenium on the metabolic pathways of serine-to-cysteine conversion and aspartate-to-methionine conversion has been widely recognized. Through the in-depth application of proteomic technologies, we can precisely identify the specific action points of selenium in these metabolic pathways. Proteomic analysis results indicate that, among the enzymes involved in the above four pathways, the significantly altered ones include cysteine-S-conjugate β-lyase, which is significantly upregulated; S-adenosyl-L-homocysteine hydrolase, which is significantly upregulated; and cysteine synthase, which is significantly upregulated.

**Figure 12:**
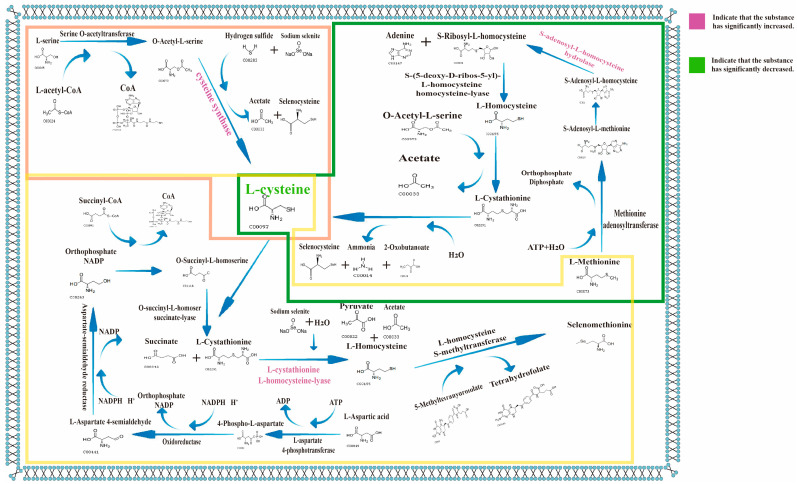
Metabolic pathway map. Figure legend: Metabolites and proteins involved in the synthesis of L-methionine from L-aspartate are represented in the yellow box; metabolites and proteins involved in the synthesis of L-cysteine from L-methionine are represented in the green box; metabolites and proteins involved in the synthesis of L-cysteine from L-serine are represented in the pink box.

Integrating metabolomics and proteomics, it is found that the effects of selenium on metabolic pathways mainly involve three pathways: serine to cysteine, methionine to cysteine, and aspartate to methionine (Figure 12). In the pathway of methionine to cysteine, we observed that S-adenosyl-L-homocysteine hydrolase (A0A0R1Y6H5|A0A0R1Y6H5_9LACO) is significantly upregulated. Since L-cysteine, the end product of this pathway, is significantly downregulated, it is possible that selenium combines with S-adenosyl-L-homocysteine hydrolase during the process where S-adenosyl-L-homocysteine is converted to S-ribosyl-L-homocysteine by the hydrolase, replacing the sulfur element in S-ribosyl-L-homocysteine and ultimately generating selenocysteine, which leads to the reduction in L-cysteine content. In the serine-to-cysteine pathway, although L-cysteine is significantly downregulated, cysteine synthase (A0A4Q7DVG0|A0A4Q7DVG0_9LACO) is significantly increased. Given that hydrogen sulfide is involved in this reaction, it suggests that selenium replaces the sulfur element in hydrogen sulfide to form selenium sulfide, which competes with hydrogen sulfide for the binding site of cysteine synthase, promoting the increase in cysteine synthase. This results in reduced cysteine synthesis and increased selenocysteine synthesis, which is also the primary reason for selenocysteine production. In the methionine synthesis pathway, the main impact of selenium is on the conversion of L-cysteine to L-methionine within this pathway. The significant increase in cysteine-S-conjugate β-lyase (C2E5J2|C2E5J2_LACJH, A0AAX3UFS7|A0AAX3UFS7_9LACO) and the significant decrease in L-cysteine, while the changes in L-homocysteine and L-cystathionine are not obvious, indicate that selenium replaces the sulfur element in L-cystathionine, promotes the synthesis of cysteine-S-conjugate β-lyase, and ultimately leads to the synthesis of selenomethionine.

It can be seen that selenium significantly affects metabolic pathways and the expression of functional proteins through regulating LAB proteins, particularly in terms of aminoacyl-tRNA synthetases and transporters. Proteomic analysis, through KEGG enrichment analysis, reveals that the role of selenium in LAB is mainly reflected in biological pathways such as antibacterial activity, antiviral activity, and transmembrane transport, with significant differences especially observed in pathological pathways like Alzheimer’s disease and Salmonella infection. In addition, GO enrichment analysis indicates that selenium promotes biological processes such as terpenoid biosynthesis, RNA synthesis, and ATPase activity. It may maintain the energy balance required for terpenoid synthesis by enhancing ATPase activity, while regulating the stability and fluidity of cell membranes, ultimately affecting the growth, function, and stress resistance of LAB. Further exploration of the selenium enrichment mechanism by combining proteomics with metabolomics shows that selenium exerts its effects mainly by influencing three metabolic pathways, serine to cysteine, methionine to cysteine, and aspartate to methionine, specifically manifested as the significant upregulation of key enzymes such as cysteine-S-conjugate β-lyase, S-adenosyl-L-homocysteine hydrolase, and cysteine synthase. Among them, in the methionine-to-cysteine pathway, the upregulation of S-adenosyl-L-homocysteine hydrolase and downregulation of L-cysteine suggest that selenium may bind to this enzyme and replace sulfur in S-ribosyl-L-homocysteine to generate selenocysteine; in the serine to cysteine pathway, the upregulation of cysteine synthase and downregulation of L-cysteine indicate that selenium replaces sulfur in hydrogen sulfide to form selenium sulfide, which competes with hydrogen sulfide for binding to this enzyme, promoting selenocysteine synthesis; in the methionine synthesis pathway, the increase in cysteine-S-conjugate β-lyase and decrease in L-cysteine (with insignificant changes in L-homocysteine and L-cystathionine) suggest that selenium replaces sulfur in L-cystathionine, promoting the synthesis of selenomethionine and ultimately leading to a reduction in L-cysteine content.

### 3.12. Correlation Analysis of Significantly Differential Metabolites Using Metabolomics Combined with Proteomics

**Figure 13:**
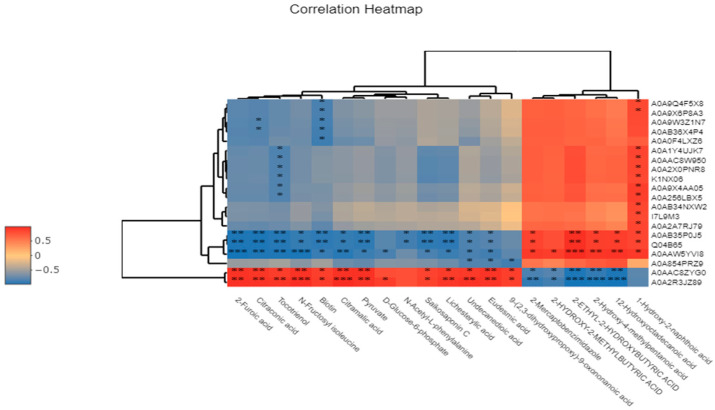
Correlation heatmap of differential metabolites and differential proteins. Figure legend: * indicates a significant correlation (*p* < 0.05); ** and *** indicate a stronger correlation.

The correlation between differential metabolites and differential proteins was calculated based on the Pearson correlation coefficient, and the correlation heatmap of the top 20 differential metabolites and differential proteins is shown in Figure 13. Pearson correlation analysis identified a strong link between key metabolites and two proteins: isoleucine-tRNA ligase and LysM domain-containing protein. Significant metabolites correlated with these proteins include 2-furoic acid, 2-ethyl-2-hydroxybutyric acid, N-fructosylisoleucine, citraconic acid, 2-hydroxy-4-methylpentanoic acid, and 12-hydroxyoctadecanoic acid. Notably, isoleucine-tRNA ligase inhibits 2-furoic acid and N-fructosylisoleucine compared to non-selenium-enriched LAB. In selenium-enriched bacteria, selenium boosts selenothiol enzyme activity, promoting 2-furoic acid accumulation, a compound involved in carbon metabolism. Furthermore, elevated levels of 2-furoic acid have the potential to inhibit the activity of isoleucine-tRNA ligase by disrupting metabolic pathways associated with this enzyme. Specifically, within amino acid synthesis pathways, 2-furoic acid may diminish the production of isoleucine through a negative feedback mechanism, thereby indirectly impeding the function of isoleucine-tRNA ligase. N-fructosylisoleucine is synthesized from isoleucine and fructose through either non-enzymatic or enzymatic glycosylation processes. In selenium-enriched LAB, selenium activates branched-chain amino acid transaminase, facilitating the conversion of isoleucine into α-ketoisocaproic acid, which subsequently enters the tricarboxylic acid cycle for energy production or the synthesis of other essential compounds. This process results in a decrease in free isoleucine, preventing its combination with fructose and consequently hindering the synthesis of N-fructosylisoleucine. Additionally, 2-ethyl-2-hydroxybutyric acid and isoleucine-tRNA ligase exhibit a mutually enhancing interaction. As an intermediary metabolite in amino acid metabolism, 2-ethyl-2-hydroxybutyric acid influences isoleucine synthesis by modulating the production of acetyl-CoA. In selenium-enriched LAB, selenium enhances acetyl-CoA synthesis, thereby promoting isoleucine production and subsequently activating isoleucine-tRNA ligase activity. This cascade of biochemical events results in an elevated synthesis of 2-ethyl-2-hydroxybutyric acid.

Citraconic acid and N-fructosylisoleucine exert a promoting effect on the LysM domain-containing protein. In the metabolic environment of selenium-enriched LAB, citraconic acid, as an important intermediate metabolite, shows a significant increase in concentration. It may activate the metabolic network related to cell wall metabolism by enhancing pyruvate-derived pathways and promoting the synthesis of peptidoglycan precursors, thereby upregulating the expression level of the LysM domain-containing protein, which is closely related to peptidoglycan recognition and regulation. Meanwhile, selenium further stabilizes the structure and function of the LysM protein by regulating antioxidant status and protein translation efficiency, leading to its significant upregulation at the proteomic level. This indicates that citraconic acid has a clear promoting effect on LysM domain-containing proteins under selenium-enriched conditions. At the metabolomic level, 2-hydroxy-4-methylpentanoic acid and 2-ethyl-2-hydroxybutyric acid are both derivatives of branched-chain amino acid catabolism. The accumulation of such metabolites under selenium-enriched conditions may indicate that cells tend to enhance energy metabolism and fatty acid oxidation pathways rather than being used for the construction of cell wall components. This metabolic tendency is inconsistent with the peptidoglycan metabolic function involved in the LysM domain-containing protein, which may thus form a metabolic competition, leading to the inhibition of the expression of this protein. In addition, the increase in these two metabolites may also activate membrane-associated stress response proteins, indirectly downregulating the expression of LysM protein to redistribute resources. 12-hydroxyoctadecanoic acid, as a hydroxylated fatty acid, is generally associated with lipid metabolism and membrane stability regulation in selenium-enriched LAB. Its accumulation is believed to be involved in the configurational remodeling of the cell membrane, helping to maintain membrane fluidity and integrity. However, the enhancement of cell membrane structure may, to a certain extent, reduce the dependence on the dynamic regulation of peptidoglycan, thereby negatively regulating cell wall metabolism-related proteins, including the LysM domain-containing protein. Meanwhile, lipid remodeling may further reduce the expression demand for LysM protein by inhibiting signaling pathways related to cell wall degradation (such as autolysin activity). Conversely, N-fructosylisoleucine is a glycosylated derivative formed by non-enzymatic reactions between amino acids and carbohydrates. Its significant increase in selenium-enriched LAB may represent a metabolic adaptation under eutrophic conditions. Its accumulation not only reflects the state of amino acid reserves but may also act as a signaling molecule to activate extracellular polysaccharide synthesis and cell wall repair pathways, thereby promoting the expression of LysM domain-containing proteins. In addition, this molecule may enhance the demand for peptidoglycan remodeling proteins by affecting intracellular osmotic pressure and stress responses, leading to the upregulation of LysM domain-containing proteins at the proteomic level.

Selenium influences LAB by modulating metabolites and protein expression. It affects isoleucine-tRNA ligase and LysM proteins by altering the metabolic network, enhancing antioxidant reactions, and promoting 2-furoic acid accumulation. Selenium also activates acetyl-CoA and isoleucine synthesis, interacting with 2-ethyl-2-hydroxybutyric acid to activate enzymes. Citraconic acid and N-fructosylisoleucine boost LysM protein expression by aiding peptidoglycan precursor synthesis, while 2-hydroxy-4-methylpentanoic acid and 12-hydroxyoctadecanoic acid inhibit it by affecting energy metabolism and membrane stability. Taken together, selenium subtly regulates the metabolic pathways and protein expression of LAB through the regulation of metabolites, exhibiting a dual effect.

## 4. Conclusions

This study conducted a comprehensive evaluation of the selenium-enriching capacity and probiotic properties of 50 strains of LAB from Xinjiang, and obtained the following key findings:

Screening and optimization of selenium-enriching strains: Through tolerance tests, eight strains with a selenium enrichment rate exceeding 80% were identified. Their optimal growth and enrichment conditions were determined as 37 °C, 2% sodium chloride, and pH 6.0 in MRS medium, laying a foundation for subsequent functional studies.

Enhanced LAB probiotic characteristics: Selenium enrichment significantly improved the antioxidant activity (DPPH and ABTS free radical scavenging) and gastrointestinal fluid tolerance of the strains, among which strain 41 performed the best, indicating its potential in the application of functional foods.

SEM-EDX analysis confirmed the presence of nano-scale selenium on the cell surface. Quantitative analysis showed that selenium was mainly distributed in selenoproteins (68.94%), followed by nucleic acids and polysaccharides, indicating that selenoproteins are the main storage form of organic selenium in these strains.

Metabolomic and proteomic studies revealed that, due to the chemical similarity between selenium and sulfur, selenium competitively binds to key enzymes such as cysteine-S-conjugate β-lyase, S-adenosyl-L-homocysteine hydrolase, and cysteine synthase to promote the conversion of serine and methionine to selenocysteine, and aspartate to selenomethionine, thereby regulating amino acid metabolic pathways. This explains the significant decrease in cysteine and the differences in protein expression.

The correlation analysis between metabolomics and proteomics indicated that selenium affects the functions of isoleucine-tRNA ligase and LysM protein by regulating metabolic networks, enhancing antioxidant responses, and promoting the accumulation of 2-furoic acid. This process activates the synthesis of acetyl-CoA and isoleucine, and synergizes with 2-ethyl-2-hydroxybutyric acid to activate related enzyme systems. Conversely, citraconic acid and N-fructosylisoleucine contribute to the expression of LysM protein by promoting the synthesis of peptidoglycan precursors. In contrast, 2-hydroxy-4-methylvaleric acid and 12-hydroxyoctadecanoic acid inhibit the expression of this protein by affecting energy metabolism and membrane stability. Ultimately, selenium achieves a precise regulation of metabolic pathways and protein expression in LAB through these two mechanisms.

Future research on the metabolic mechanism of selenium enrichment in LAB, if combined with selenium isotope technology and the metabolomic and proteomic analysis of selenium-enriched LAB at different growth and development stages, will help clarify the selenium enrichment process more accurately.

In summary, this study clarifies the selenium-enriching mechanism and probiotic potential of characteristic LAB from Xinjiang, providing a theoretical basis for the development of selenium-enriched functional foods and the utilization of regional microbial resources.

## Figures and Tables

**Table 1:** Color change of selenium-enriched LAB.

Strain Number Sodium Selenite Concentration	0	20	40	60	80	100
1	-	+	++	+++	++++	++++
2	-	+	++	+++	++++	++++
3	-	++	++	+++	++++	++++
4	-	+	++	+++	++++	++++
5	-	+	++	+++	++++	++++
6	-	+	++	+++	++++	++++
7	-	+	++	+++	++++	++++
8	-	+	++	+++	++++	++++
9	-	++	+++	++++	++++	++++
10	-	+	++	+++	++++	++++
11	-	+	++	+++	++++	++++
12	-	+	++	+++	++++	++++
13	-	++	+++	++++	++++	++++
14	-	+	++	+++	++++	++++
15	-	+	++	+++	++++	++++
16	-	+	++	+++	++++	++++
17	-	+	++	+++	++++	++++
18	-	+	++	+++	++++	++++
19	-	+	++	+++	++++	++++
20	-	+	++	+++	++++	++++
21	-	++	+++	++++	++++	++++
22	-	+	++	+++	++++	++++
23	-	+	++	+++	++++	++++
24	-	+	++	+++	++++	++++
25	-	+	++	+++	++++	++++
26	-	+	++	+++	++++	++++
27	-	+	++	+++	++++	++++
28	-	++	+++	++++	++++	++++
29	-	++	+++	++++	++++	++++
30	-	+	++	+++	++++	++++
31	-	+	++	+++	++++	++++
32	-	++	+++	++++	++++	++++
33	-	+	++	+++	++++	++++
34	-	+	++	+++	++++	++++
35	-	++	++	+++	++++	++++
36	-	+	++	+++	++++	++++
37	-	+	++	+++	++++	++++
38	-	+	++	+++	++++	++++
39	-	+	++	+++	++++	++++
40	-	+	++	+++	++++	++++
41	-	+	++	+++	++++	++++
42	-	+	++	+++	++++	++++
43	-	+	++	+++	++++	++++
44	-	+	++	+++	++++	++++
45	-	+	++	+++	++++	++++
46	-	+	++	+++	++++	++++
47	-	+	++	+++	++++	++++
48	-	+	++	+++	++++	++++
49	-	+	++	+++	++++	++++
50	-	+	++	+++	++++	++++

- Normal, + Slightly red, ++ Pink, +++ Red, ++++ Deep red.

## Data Availability

The original contributions presented in this study are included in the article. Further inquiries can be directed to the corresponding author.
